# Efficient infection of non-human primates with purified, cryopreserved *Plasmodium knowlesi* sporozoites

**DOI:** 10.1186/s12936-022-04261-z

**Published:** 2022-08-27

**Authors:** Sumana Chakravarty, Melanie J. Shears, Eric R. James, Urvashi Rai, Natasha KC, Solomon Conteh, Lynn E. Lambert, Patrick E. Duffy, Sean C. Murphy, Stephen L. Hoffman

**Affiliations:** 1 Sanaria, Inc, 9800 Medical Center Drive, Suite A209, Rockville, MD 20850 USA; 2 Department of Laboratory Medicine and Pathology, University of Washington, Seattle, WA USA; 3 Washington National Primate Research Center, University of Washington, Seattle, WA USA; 4 Laboratory of Malaria Immunology and Vaccinology, NIAID/NIH, Bethesda, USA; 5 Department of Microbiology, University of Washington, Seattle, WA USA

## Abstract

**Background:**

*Plasmodium falciparum* (Pf) sporozoite (SPZ) vaccines are the only candidate malaria vaccines that induce > 90% vaccine efficacy (VE) against controlled human malaria infection and the only malaria vaccines to have achieved reproducible VE against malaria in adults in Africa. The goal is to increase the impact and reduce the cost of PfSPZ vaccines by optimizing vaccine potency and manufacturing, which will benefit from identification of immunological responses contributing to protection in humans. Currently, there is no authentic animal challenge model for assessing *P. falciparum* malaria VE. Alternatively, *Plasmodium knowlesi* (Pk), which infects humans and non-human primates (NHPs) in nature, can be used to experimentally infect rhesus macaques (*Macaca mulatta*) to assess VE.

**Methods:**

Sanaria has, therefore, produced purified, vialed, cryopreserved PkSPZ and conducted challenge studies in several naïve NHP cohorts. In the first cohort, groups of three rhesus macaques each received doses of 5 × 10^2^, 2.5 × 10^3^, 1.25 × 10^4^ and 2.5 × 10^4^ PkSPZ administered by direct venous inoculation. The infectivity of 1.5 × 10^3^ PkSPZ cryopreserved with an altered method and of 1.5 × 10^3^ PkSPZ cryopreserved for four years was tested in a second and third cohort of rhesus NHPs. The lastly, three pig-tailed macaques (*Macaca nemestrina*), a natural *P. knowlesi* host, were challenged with 2.5 × 10^3^ PkSPZ cryopreserved six years earlier.

**Results:**

In the first cohort, all 12 animals developed *P. knowlesi* parasitaemia by thick blood smear, and the time to positivity (prepatent period) followed a non-linear 4-parameter logistic sigmoidal model with a median of 11, 10, 8, and 7 days, respectively (r^2^ = 1). PkSPZ cryopreserved using a modified rapid-scalable method infected rhesus with a pre-patent period of 10 days, as did PkSPZ cryopreserved four years prior to infection, similar to the control group. Cryopreserved PkSPZ infected pig-tailed macaques with median time to positivity by thin smear, of 11 days.

**Conclusion:**

This study establishes the capacity to consistently infect NHPs with purified, vialed, cryopreserved PkSPZ, providing a foundation for future studies to probe protective immunological mechanisms elicited by PfSPZ vaccines that cannot be established in humans.

**Supplementary Information:**

The online version contains supplementary material available at 10.1186/s12936-022-04261-z.

## Background

Radiation-attenuated and chemo-attenuated *Plasmodium falciparum* (Pf) sporozoite (SPZ) vaccines have protected subjects against controlled human malaria infection and natural exposure to *P. falciparum* in Africa in numerous clinical trials [1–19]. As the first candidate PfSPZ vaccines move toward licensure, the goal is to develop subsequent generation vaccines with increased potency, durability, and breadth of protection and decreased cost of goods. Understanding the induction of protective immunity and the mechanisms of protective immunity will be fundamental to achieving the next generation goals. Studies in humans have demonstrated that induction of protective immunity is significantly improved by administration of PfSPZ by direct venous inoculation, indicating induction takes place optimally in tissues directly accessible to venous circulation [1, 3]. Furthermore, data generated in mice and non-human primates (NHPs) during the past 35 years indicate that the effector mechanism of protection takes place in the liver [1, 20–23]. During the past decades several studies have reported on human immune responses in serum, plasma, and peripheral blood mononuclear cells that have in some cases been associated with protective immunity [3, 11]. However, these are almost certainly non-mechanistic surrogates/biomarkers of protective immunity, because we cannot systematically sample the liver, liver-draining lymph nodes and spleen in humans.

A model system in NHP that will allow for detailed immunologic analysis at the site of SPZ-based vaccine immunity, and simultaneous determination of vaccine efficacy through controlled challenge, is therefore necessary. *Plasmodium knowlesi* (Pk) is the only *Plasmodium* sp. malaria parasite that naturally infects humans and is routinely used to infect rhesus macaques (*Macaca mulatta*) in an experimental setting. Thus, it provides an excellent platform for investigating critical questions in malaria vaccinology. This model has been used for development of subunit and vectored vaccine strategies against malaria, and to demonstrate the critical role of CD8^+^ T cells in protection [24–26].

*P. knowlesi* malaria is also a public health problem, primarily in populations living or working in forested areas in Southeast (SE) Asia where the NHPs that carry this parasite live and in recent years, malaria in humans due to *P. knowlesi* has been reported extensively [27–29]. A NHP model for studying *P. knowlesi* malaria may, therefore, also be beneficial for investigating the biology and transmission of this zoonotic parasite.

To date, only a few studies [30–32] have utilized *P. knowlesi* sporozoites in NHPs to study mechanisms of protective immunity, and none have utilized Sanaria’s purified, cryopreserved, vialed PkSPZ. Capacity to produce and cryopreserve purified, infectious PfSPZ enabled development of a comparable system for PkSPZ. This is the first NHP study demonstrating infectivity of purified, cryopreserved PkSPZ in rhesus macaques, and the infection of a natural NHP host, the pig-tailed macaque (*Macaca nemestrina*).

## Methods

### Parasites and mosquitoes

To make purified, cryopreserved PkSPZ, mosquitoes infected with *P. knowlesi* H strain were kindly provided by Dr. Patrick Duffy (LMIV, NIAID, NIH). The mosquitoes were generated as follows: *P. knowlesi*-infected red blood cells (NIH stock) were used to infect splenectomized, anesthetized rhesus macaques. Subsequent feeding of *Anopheles dirus* mosquitoes on the infected animals was performed as described [33].

### Production of purified, cryopreserved PkSPZ

PkSPZ were hand-dissected from mosquito salivary glands and purified from mosquito salivary gland material using methods developed for PfSPZ [34]. Cryopreservation followed procedures used to routinely manufacture PfSPZ products, with modifications for a rapid-scalable method.

### Infection of rhesus macaques

Rhesus macaques (*Macaca mulatta*) were housed at BIOQUAL, Inc. at its Rockville facilities in accordance with the recommendations of the Association for Assessment and Accreditation of Laboratory Animal Care (AAALAC) International Standards and with the recommendations in the NIH Guide for the Care and Use of Laboratory Animals of the USA. Bioqual is accredited by the AAALAC (file #624) and holds an Assurance on file with the National Institutes of Health, Office of Protection from Research Risks as required by the US Public Health Service Policy on Humane Care and Use of Laboratory Animals. The Institutional Animal Use and Care Committee of BIOQUAL approved these experiments. When immobilization was necessary, the animals were sedated intramuscularly with 10 mg/kg of Ketamine HCl (Parke-Davis, Morris Plains N.J.) before any direct handling or procedures. Animals were housed in an air-conditioned facility with an ambient temperature of 21–25 °C, a relative humidity of 40–60% and a 12 h light/dark cycle. Animals were socially housed when possible or individually housed if no compatible pairing could be found. The animals were housed in suspended stainless-steel wire-bottomed 6 sq ft cages and provided with a commercial primate diet and fresh fruit and vegetables twice daily with water freely available at all times. Social housing, toys, foraging equipment and mirrors were provided. Animals were monitored at least twice daily for behaviour, food intake, activity, and overall health by trained technicians. In order to minimize the use of NHPs, who had experienced non-malaria related interventions in previous studies, were used. The cohort used in the first study were a kind gift from Dr. Robert Seder (VRC, NIH).

Rhesus macaques were stratified into comparable groups based on age, weight, and sex of the animals. Purified, cryopreserved PkSPZ, which had been cryopreserved 1 to 4 years earlier were thawed, diluted, and injected in a volume of 0.5 mL in a manner analogous to administration of Sanaria’s PfSPZ products to humans by direct venous inoculation [1, 2]. Animals were sedated with ketamine or telazol for all technical procedures. Ketamine was given IM in amounts necessary for short-term procedures such as blood drawing. The study endpoint was the detection of parasites on thick blood smear preparations and parasite development up to 0.8-5% parasitaemia at which time animals were immediately treated.

### **Detection of*Plasmodium knowlesi*parasites in blood**

#### Blood collection and handling

Animals housed at BIOQUAL were bled from the femoral artery and 1–2 mL of whole blood was collected into a labeled ethylenediaminetetraacetic acid (EDTA) vacutainer tube. The vacutainer was inverted gently several times to ensure adequate anticoagulation. Whole blood specimens were then transported to Sanaria in insulated containers containing conditioned ice packs or at ambient temperature. Blood specimens were processed immediately for qRT–PCR by aliquoting and mixing 50 µL of EDTA-anticoagulated whole blood into each of two tubes containing 2 mL NucliSENS lysis buffer (bioMérieux Inc., Marcy-lÉtoile, France). The lysis buffer tubes were then frozen and stored at − 80 °C until shipping. Small amounts of the anti-coagulated blood were used to prepare blood smear slides.

#### Blood smear assessments

*P. knowlesi* blood stages were detected by examining Giemsa-stained thick and thin blood smears at 1000× magnification. Thick blood smear slides were prepared as described [5]. Briefly, A microscope slide was placed over a template with a 1 cm × 2 cm rectangle and ten microliters of blood was evenly distributed over the entire 1 cm × 2 cm rectangular area with a micropipette. The slide was dried and stained with 10% Giemsa (Giemsa Stain cat#GS500-500ML Sigma Aldrich], pH7.2, for 10 ± 2 min. On a light microscope with a 100X objective, using vertical passes that are 1 cm long and the width of the high-power field (HPF), the number of asexual parasites observed in the number of passes appropriate to the objective lens (0.22 mm) were recorded. Each HPF on this thick smear is 0.11 µl (on a standard microscope at 1,000 magnification). The theoretical limits of detection were calculated as follows.

**Table:** 

Area of template	10 × 20 = 200 mm ^2^
Volume of blood	10 mm ^3^
Depth of smear	10 ÷ 200 = 0.05 mm
Objective lens	0.22 mm
1 pass under objective lens	0.22 mm × 10 mm × 0.05 mm = 0.11 mm ^3^ = 0.11 µL
Number of passes	5
Total volume read over 5 passes (µL)	5 × 0.11 µL = 0.55 µL
Theoretical density at 2 parasites detected	2 ÷ 0.55 = 3.64 parasites / µL

The preparations were examined daily, starting on day 5 post-infection, until parasitaemia was confirmed on two consecutive days. This was followed by thin-smear assessments for quantitative scores of parasitaemia development, until anti-malaria treatment was initiated. The pre-patent period in rhesus was defined as the period between inoculation of PkSPZ and appearance of the first positive in a thick blood smear. An animal was considered positive if one or more unambiguous asexual parasites were observed by two readers, on a TBS.

#### Plasmodium multiplex PCR

Multiplex PCR was performed by Dr. Gary Fahle (Department of Laboratory Medicine, NIH Clinical Center) in support of rhesus macaque infections at BIOQUAL [3].

#### Plasmodium knowlesi qRT–PCR

The University of Washington Malaria Molecular Diagnostic Laboratory performed nucleic acid extraction on an Abbott m2000sp and *Plasmodium* 18 S rRNA qRT–PCR on an Abbott m2000rt as previously described [35]. The standard clinical assay was performed including multiplexed pan-*Plasmodium*, *P. falciparum*-specific, and host mRNA control channels. Absolute copy number quantification was estimated from the pan-*Plasmodium* 18 S rRNA channel using an Armored RNA calibrator curve (Asuragen) in malaria-negative whole blood. The estimated copy numbers were reported as copies/mL of whole blood.

### Infection of pig-tailed macaques

Pig-tailed macaques *(Macaca nemestrina)* were housed at the Washington National Primate Research Center (WaNPRC) in Seattle. All procedures were conducted in accordance with an approved University of Washington Institutional Animal Care and Use Committee Protocol, and animals were cared for in accordance with the NIH Guide for the Care and Use of Laboratory Animals. The environment at the WaNPRC is maintained at 23–25 °C, 30–70% relative humidity, with 10–15 air changes hourly and a 12 h/12hr light/dark cycle. All animals were housed socially in pairs, fed an appropriate diet, and given free access to water. Environmental enrichment was provided in accordance with WaNPRC Behavioral Management Services standing operating procedures. When immobilization was necessary, the animals were sedated intramuscularly with ketamine HCl before procedures. Animals used in this study were malaria-naïve males. Infection was performed by direct venous inoculation of 2.5 × 10^3^ PkSPZ cryopreserved 6 years earlier, into the saphenous vein of ketamine-sedated animals. PkSPZ were shipped, stored, thawed, diluted, and administered in strict accordance with Sanaria protocols.

### **Detection of*P. knowlesi*parasites in pig-tailed macaque blood**

To assess blood stage infection, animals were monitored daily starting Day 6 post infection. Cage-side monitoring was performed by trained technicians to assess activity level, lethargy, appetite, urine and fecal output. Blood was sampled from the saphenous vein to make two replicate thin Giemsa-stained blood smears per animal per day. Approximately 100 µL of blood was collected onto Whatman Protein Saver cards (#903TM) to create two dried blood spots for *Plasmodium* 18 S rRNA qRT–PCR analysis performed as above. The pre-patent period in pig-tailed macaques was defined as the period between inoculation of PkSPZ and appearance of the first positive in a thin blood smear. Thin blood smears were assessed at 1000X magnification and approximately 50 high-powered fields were examined per animal per day. A positive thin blood smear was called when two unambiguous parasites were observed in 50 fields (estimated limit of detection ~ 0.02% parasitaemia). Infected animals were treated with oral chloroquine (10 mg/kg for three days) upon reaching 0.8-2% blood stage parasitaemia, or after two weeks of blood smear positivity, whichever was first. Parasite clearance following anti-malarial treatment was defined as two sequential negative qRT–PCR results.

### Non-linear regression

Using data from our first dose-response study, the median pre-patent period was plotted against the dose of PkSPZ in MyCurveFit (online curve-fitting software https://mycurvefit.com/). A non-linear 4-parameter logistic sigmoidal model was the best fit with an R^2^ of 1.

## Results

### First attempts to infect rhesus macaques with purified cryopreserved PkSPZ

The first infection of rhesus with Sanaria’s vialed, purified, cryopreserved PkSPZ was demonstrated in 2010 and is now reported in Additional file 1: Table S1.

### Dose response of purified, cryopreserved PkSPZ in NHPs with no previous exposure

**Fig. 1:**
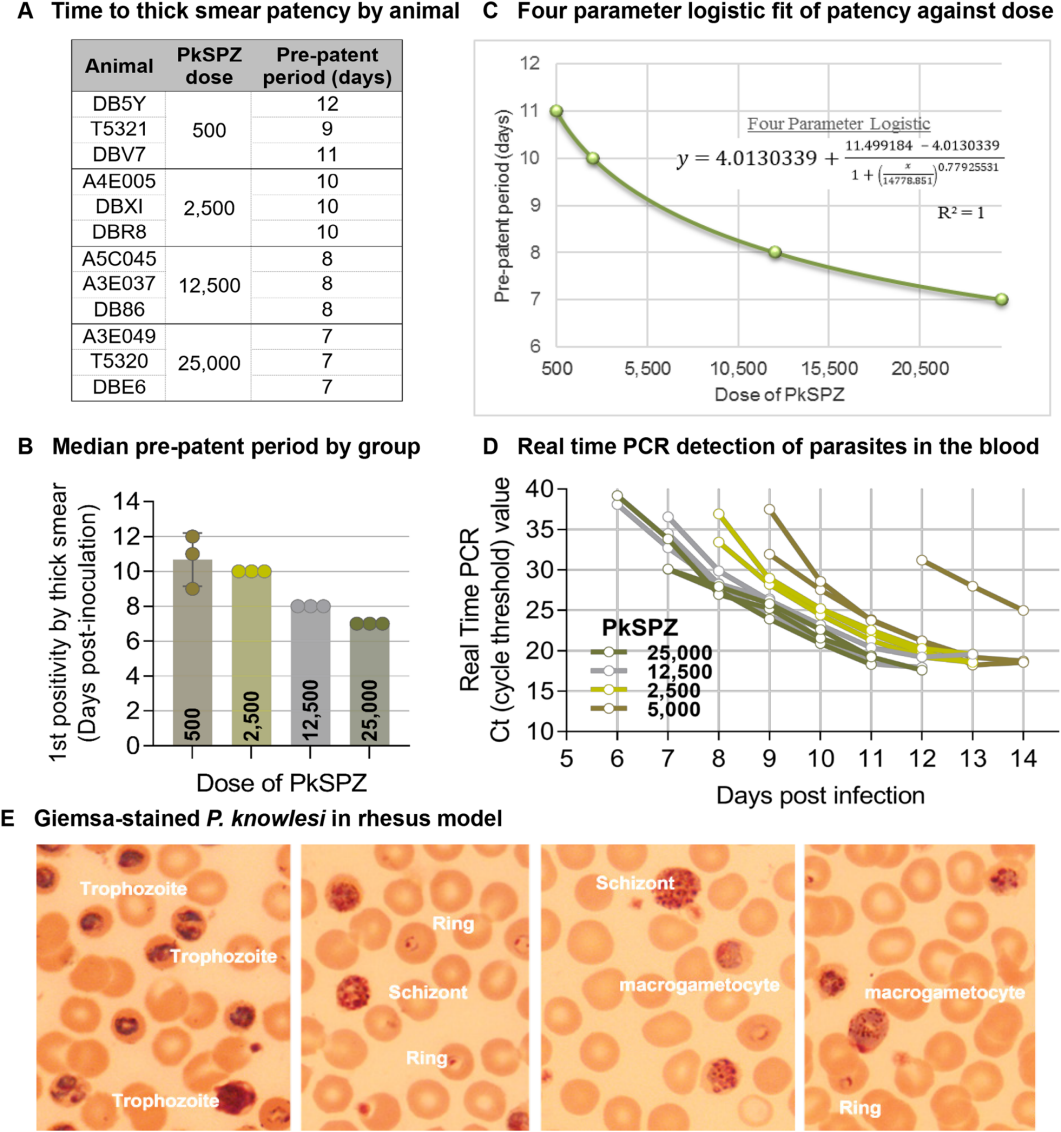
Development of parasitaemia in rhesus macaques inoculated with varying doses of purified, vialed, cryopreserved PkSPZ. **A** Pre-patent periods by animal as defined by first appearance of detectable parasites in thick blood smear preparations. **B** Median pre-patent periods in each dose group. **C** Four parameter logistic fit of pre-patent periods plotted against PkSPZ dose (R^2^ = 1). **D** NIH multiplex PCR analysis of parasitaemia development in blood. **E** Photomicrographs of asexual and sexual *P. knowlesi* stages in Giemsa-stained thin blood smears

To determine the minimum dose of PkSPZ required for 100% infection, in the first study cohort, four groups of three rhesus macaques each were infected with varying numbers of purified, cryopreserved PkSPZ, and infectivity assessed by thick and thin Giemsa-stained blood smear and real-time PCR. 100% of animals were infected at all doses tested (5 × 10^2^, 2.5 × 10^3^, 1.25 × 10^4^, 2.5 × 10^4^ PkSPZ). The median pre-patent periods were 11, 10, 8 and 7 days for the 5 × 10^2^, 2.5 × 10^3^, 1.25 × 10^4^, and 2.5 × 10^4^ dose groups, respectively (Fig. 1A, B). Five hundred PkSPZ were 100% infective but resulted in longer and more variable pre-patent periods in the three animals in the group (Fig. 1A). A non-linear 4-parameter logistic sigmoidal model was the best fit when numbers of PkSPZ inoculated (Y axis) were plotted against pre-patent period (median or geometric mean; X axis), with an R^2^ of 1 (Fig. 1C).

The kinetics of PkSPZ in blood of infected animals was analyzed by a multiplex malaria PCR ([7–9]; limit of detection defined as 700 parasites/mL of whole blood). The cycle threshold (Ct) values are plotted in Fig. 1D. The assay was not standardized enough to generate a reference curve specifically for *P. knowlesi*. However, parasites were detected in blood about a day earlier than positivity in thick smears, and the trends with the Ct values confirmed that animal DB5Y was the slowest to develop parasitaemia, with PCR detection possible only by day 12. Asexual and sexual stages of *P. knowlesi* were detected in thin blood smears (Fig. 1E).

### Effect of modifying cryopreservation method for efficiency and scalability, on infectivity of PkSPZ

**Fig. 2:**
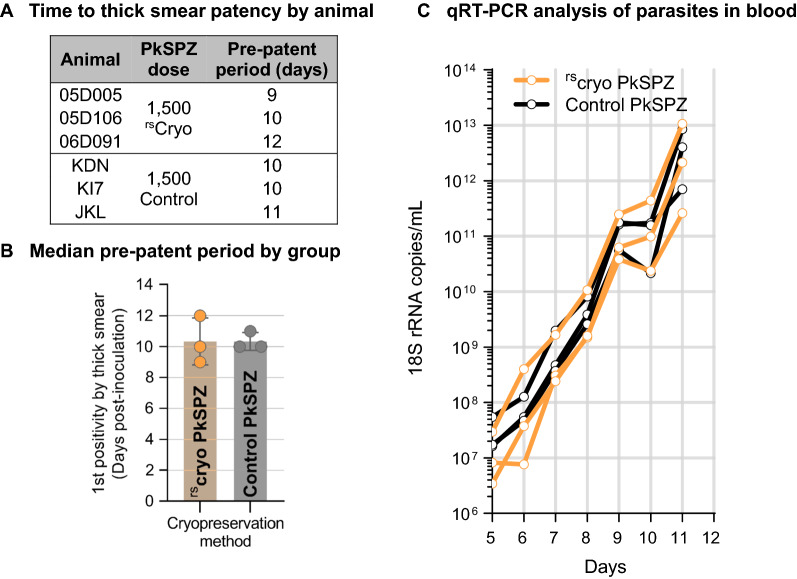
Development of parasitaemia in rhesus macaques inoculated with vialed purified PkSPZ cryopreserved using two different methods.** A**, **B** Pre-patent periods by animal and group as defined by first appearance of detectable parasites in thick blood smear preparations. **C** Change in *Plasmodium* 18 S rRNA copies in blood post-infection by 18 S rRNA qRT–PCR performed at the University of Washington

The second cohort study was designed to compare the infectivity of PkSPZ cryopreserved by standard methods used for Sanaria’s PfSPZ Vaccine versus a rapid, scalable cryopreservation (^rs^cryo) method developed at Sanaria. Groups of three rhesus macaques were infected with 1.5 × 10^3^ purified, cryopreserved PkSPZ produced by each method from the same batch of PkSPZ infected mosquitoes, and infectivity assessed by thick and thin Giemsa-stained blood smear and real-time PCR. A dose of 1.5 × 10^3^ PkSPZ was sufficient to infect 100% of the NHPs (Fig. 2A–C). The first animal to become patent had been injected with ^rs^cryo PkSPZ (Fig. 2A). The median pre-patent period for the ^rs^cryo PkSPZ group was identical to the control PkSPZ group at 10 days. When the 4-Parameter Log sigmoidal model generated in Fig. 1C was used to predict the pre-patent period for this study with all animals inoculated with 1.5 × 10^3^ PkSPZ, the calculated pre-patency of 10.4 days was very close to the observed pre-patency (geometric mean 10.26 and 10.32; median 10 and 10, respectively) with PkSPZ cryopreserved using rapid-scalable or control methods. There was no difference in the 18 S rRNA biomarker densities between groups on Days 5, 6, or 7 post-infection (unpaired *t*-tests). The 18 S rRNA biomarker increased with comparable kinetics for both groups with rising densities on all but one day (Fig. 2C), consistent with the ~ 24-hour lifecycle of *P. knowlesi* in erythrocytes.

### Infectivity of PkSPZ four years after cryopreservation

**Fig. 3:**
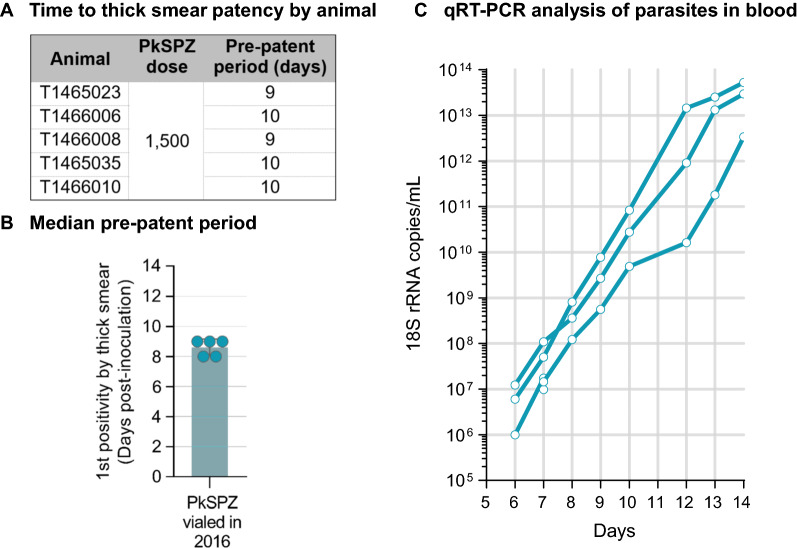
Development of parasitaemia in rhesus macaques inoculated in 2020 with PkSPZ vialed and cryopreserved in 2016.** A**, **B** Pre-patent periods by animal and group as defined by first appearance of detectable parasites in thick blood smear preparations. **C**. Change in *Plasmodium* 18 S rRNA copies in blood post-infection by 18 S rRNA qRT–PCR performed at the University of Washington

Next, in the third cohort, PkSPZ vialed in 2016 were used to challenge five rhesus macaques in 2020. A dose of 1.5 × 10^3^ PkSPZ was sufficient to infect 100% of the NHPs, and the PkSPZ showed no diminution in activity as evidenced by the resulting pre-patent periods in infected animals (Fig. 3 A-C). The median pre-patent period for this group was 10 days and the geometric mean was 9.6 days, very similar to the pre-patent periods observed for this PkSPZ dose in study cohort two described earlier. 18 S rRNA was detected in 3/5 animals on Day 6 and 5/5 animals on Day 7; 18 S rRNA qRT–PCR was not performed on Day 5. Comparing the Day 7 18 S rRNA data for this cohort (2020 challenge) versus the 2016 challenge cohort, there was a statistically significant difference in the 18 S rRNA densities with slightly lower Day 7 densities in the 2020 challenge group compared to the 2016 challenge (p = 0.048, two-tailed unpaired t-test, Fig. 3C). However, the rise in densities thereafter was not different than seen in the earlier study, despite the fact that animal infection, blood collection, and the qRT–PCR for the two cohorts were performed four years apart.

### Infectivity of cryopreserved PkSPZ in pig-tailed macaques with no previous exposure

**Fig. 4:**
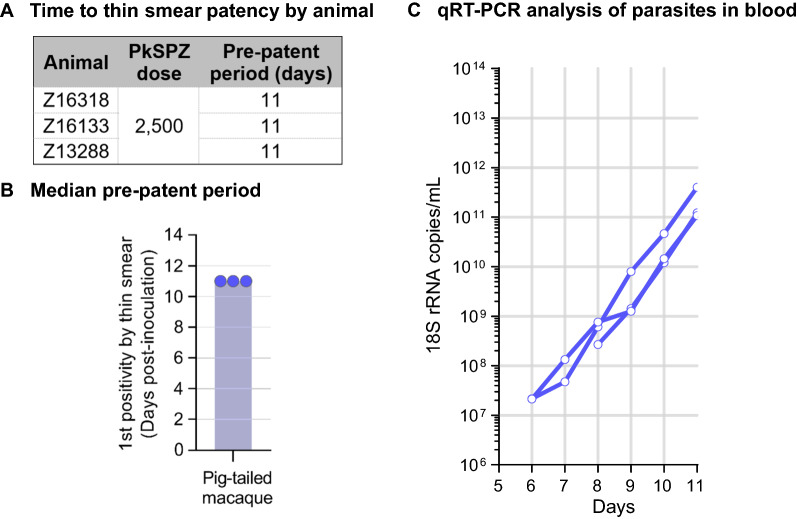
Development of parasitaemia in pig-tailed macaques.** A**, ** B** Pre-patent periods by animal and group as defined by first appearance of detectable parasites in thin blood smear preparations **C** Change in *Plasmodium* 18 S rRNA copies in blood post-infection by 18 S rRNA qRT–PCR performed at the University of Washington

Finally, a pilot study to test the infectivity of cryopreserved PkSPZ in a natural host, the pig-tailed macaque was performed. A dose of 2.5 × 10^3^ PkSPZ was selected for this pilot study since it gave highly reproducible pre-patent periods in rhesus macaques (Fig. 1). This study was performed with PkSPZ that were cryopreserved six years prior. All three pig-tailed macaques developed blood stage infections with a time to patency by thin Giemsa-stained blood smear of 11 days (Fig. 4A, B). *Plasmodium* 18 S rRNA qRT–PCR became positive in the blood starting at 6–8 days post PkSPZ infection and copies steadily increased until animals became patent by blood smear (Fig. 4C).

## Discussion and conclusion

Purified, cryopreserved PkSPZ produced using Sanaria’s platform technology were infective to rhesus macaques and pig-tailed macaques when administered 1 (cohort 1), 2 (cohort 2), 4 (cohort 3), or 6 (pig-tailed macaques) years after cryopreservation. In study cohorts one, two and three, 100% of rhesus macaques in all dose groups became parasitaemic, and erythrocytic stage of *P. knowlesi* were detected in thick blood smears and by RT–PCR. Modification of the cryopreservation method for increased efficiency and scalability did not adversely affect PkSPZ potency. Cryopreservation for four years similarly did not adversely affect PkSPZ infectivity. In the final pilot cohort, all three pig-tailed macaques in the single dose group tested became parasitaemic and blood stage *P. knowlesi* were detected in thin blood smears and by RT–PCR.

In the first study cohort, the dose response followed a four-parameter logistic fit (r^2^ = 1) in the time to develop detectable parasitaemia (pre-patent period by thick smear) in the four groups of rhesus macaques. The median pre-patent periods were 11, 10, 8, and 7 days for the 5 × 10^2^, 2.5 × 10^3^, 12.5 × 10^4^, and 2.5 × 10^4^ PkSPZ groups, respectively (r^2^ = 1, p = 0.01). Inoculation of 5 × 10^2^ PkSPZ was sufficient to impart *P. knowlesi* erythrocytic stage infection in 100% (3/3) of recipients. However, in these animals the pre-patent period was longer than the other groups, and less uniform among the three animals. In study cohorts two and three, the time to develop detectable parasitaemia for 1.5 × 10^3^ PkSPZ in rhesus macaques was consistently 10 days. In the final pilot in pig-tailed macaques, the median pre-patent period by thin blood smears for 2.5 × 10^3^ PkSPZ was 11 days. The limit of detection for thin smears is estimated to be 11-times less sensitive than for thick blood smears [36]. Thus, considering this difference in sensitivity, the pre-patent period in pig-tailed macaques was largely consistent with that for rhesus macaques with the equivalent PkSPZ dose.

A literature review to assess how the infectivity of cryopreserved PkSPZ reported here compared against infections in various NHP species induced by freshly dissected *P. knowlesi* sporozoites administered by needle and syringe or mosquito bites, is described in Additional file 2: Table S2. The prepatent period reported in most studies ranged from 9 to 12 days in two major NHP hosts used for *P. knowlesi* studies, rhesus macaques and *Saimiri boliviensis*. In general, the prepatent periods observed after fresh *P. knowlesi* sporozoite inoculation correlated with dose; the higher the dose of *P. knowlesi* sporozoites, the shorter the prepatent period. In these studies [25, 37–39], doses of 50, 100–200, and 1.1 × 10^6^ *P. knowlesi* sporozoites resulted in pre-patent periods of 11–16, 7–10, and 6 days respectively. In one study, *P. knowlesi* sporozoites were harvested from the salivary glands of *An. dirus* mosquitoes into 50% FBS-PBS and stored frozen in liquid nitrogen vapor phase. When the frozen *P. knowlesi* sporozoites were inoculated within 7–11 years after freezing, prepatent periods were in the range of 6–10 days with 4.5 × 10^4^ – 1.25 × 10^5^ SPZ inoculated [39]. In a few studies [38, 40] using mosquito bite inoculation of PkSPZ in *M. mulatta, Aotus*, or *S. boliviensis* shorter prepatent periods of 7–9 days were observed with 2–4 mosquito bites, although it is difficult to estimate the exact number of PkSPZ inoculated in these cases.

Over multiple clinical trials, direct venous inoculation (DVI) of 3,200 aseptic, purified cryopreserved PfSPZ (Sanaria^®^ PfSPZ Challenge) resulted in 100% infection of *P. falciparum*-malaria-naïve Americans and Europeans, and DVI of 800 PfSPZ infected 7/9 subjects [4–6, 8, 17, 26, 41–44]. The fact that 100% infection of the rhesus macaques was achieved with 5 × 10^2^ PkSPZ and 100% of pig-tailed macaques with 2.5 × 10^3^ PkSPZ, indicates PkSPZ may be more infectious to NHPs than PfSPZ is to humans. These data establish a baseline for conducting experimental challenge studies with purified, cryopreserved PkSPZ. Such studies could be critical in helping us to unravel the mechanisms of induction and the mechanisms and targets of protective immune responses after immunization with whole SPZ vaccines. Cryopreserved PkSPZ can be used to conduct in-depth analyses of immunological mechanisms and correlates of protection elicited by PkSPZ–based vaccination approaches that cannot be substantiated in humans. The data also demonstrate that a new method of cryopreservation, with improved scalability and ease of execution did not adversely affect PkSPZ infectivity, and that the cryopreserved PkSPZ do not lose potency over many years when stored in liquid nitrogen vapor phase (LNVP).

Rhesus macaques are currently the preferred model for assessing vaccines against *P. falciparum* malaria, and RTS,S, ME-TRAP and PfSPZ were all tested in rhesus macaques early in clinical development [1, 45, 46]. However, due to COVID-19 research, the US is currently facing a shortage of rhesus macaques, suggesting that identification of a suitable alternative macaque species for malaria vaccine studies may be useful. Pigtailed macaques are closely related to rhesus macaques, and are used as models to study numerous infectious diseases, including HIV [47, 48], chlamydia [49], Zika [50] and *Staphylococcus aureus* [51]. Pig-tailed macaques are notably also the natural forest host of *P. knowlesi* malaria parasites [52]. However, despite their potential, pigtailed macaques have never previously been used as a model for malaria vaccine testing. In this study, 3/3 naïve pigtailed macaques were reliably infected with a dose of 2.5 × 10^3^ PkSPZ. This preliminary data suggest that pigtailed macaques may hold promise as an alternative model for *P. knowlesi* malaria vaccination and challenge studies that could be used in a similar manner to the rhesus macaque model.

## Supplementary Information

**Additional file 1: Table S1.** Rhesus macaques with prior *P. knowlesi* exposure infected with purified, cryopreserved PkSPZ.

**Additional file 2: Table S2.** Literature review for infectivity of fresh *Plasmodium knowlesi* sporozoites introduced by direct inoculation or by mosquito bite.

## Data Availability

The dataset(s) supporting the conclusions of this article is(are) included within the article.

## Abbreviations

Pf: *Plasmodium falciparum*
SPZ: sporozoite
VE: vaccine efficacy
Pk: *Plasmodium knowlesi*
NHPs: Non-human primates
AAALAC: Association for Assessment and Accreditation of Laboratory Animal Care
EDTA: Ethylenediaminetetraacetic acid
HPF: High-power field
WaNPRC: Washington National Primate Research Center
^rs^cryo: Rapid, scalable cryopreservation
DVI: Direct venous inoculation
LNVP: Liquid nitrogen vapor phase
