# Supplementary material for: Efficient infection of non-human primates with purified, cryopreserved Plasmodium knowlesi sporozoites
Source: Malar J. 2022 Aug 27;21:247. doi: 10.1186/s12936-022-04261-z (PMC9418655; doi:10.1186/s12936-022-04261-z)
Supplement: Supplementary file 1 — Additional file 1: Table S1. Rhesus macaques with prior P. knowlesi exposure infected with purified, cryopreserved PkSPZ. [file 12936_2022_4261_MOESM1_ESM.doc]

**Table S1: Rhesus macaques with prior *P. knowlesi* exposure infected with purified, cryopreserved PkSPZ.**

| | Animal ID | Date of PkSPZ injection | Date of parasite appearance in blood by thick blood smear | Pre-patent period (days) | | --- | --- | --- | --- | | 1 | 07/12/2010 | 07/29/2010 | 17 | | 2 | 06/21/2010 | 06/29/2010 | 8 | | 3 | 06/21/2010 | 06/29/2010 | 8 | | 4 | 06/04/2010 | 06/12/2010 | 8 | | 5 | 07/21/2010 | Blood smear negative until 01 Aug 2010; inoculated with P. knowlesi infected RBC on 02 Aug 2010 (day 12) and was blood smear positive on 06 Aug 2010 | | |
| --- | --- | --- | --- | --- | --- | --- | --- | --- | --- | --- | --- | --- | --- | --- | --- | --- | --- | --- | --- | --- | --- | --- | --- | --- |

Rhesus NHPs with prior *P. knowlesi* exposure infected with purified, cryopreserved PkSPZ: The set of animals used for this had been used in other malaria-intervention studies. Thus, the prior malaria history including exposure to *P. knowlesi*, could have affected outcome. However, the goal here was to ascertain if purified, cryopreserved PkSPZ were at all infectious *in vivo*. Five rhesus macaques were used in the study, and inoculated intravenously with 5×10^3^ PkSPZ. While animals #2, #3, and #4 did become pre-patent on day 8, animal #1 took 17 days to exhibit patent parasitaemia, possibly due to reasons cited above. The outcome with animal #5 was more difficult to interpret. The animal was not parasitaemic for 11 days after PkSPZ injection, and upon subsequent inoculation with blood stage parasites on day 12, was patent 4 days later. However, the cause of the patency could be attributed to either the initial PkSPZ or later *P. knowlesi* blood stage inoculations and a definitive conclusion was not possible.
