# Supplementary material for: Efficient infection of non-human primates with purified, cryopreserved Plasmodium knowlesi sporozoites
Source: Malar J. 2022 Aug 27;21:247. doi: 10.1186/s12936-022-04261-z (PMC9418655; doi:10.1186/s12936-022-04261-z)
Supplement: Supplementary file 2 — Additional file 2: Table S2. Literature review for infectivity of fresh Plasmodium knowlesi sporozoites introduced by direct inoculation or by mosquito bite. [file 12936_2022_4261_MOESM2_ESM.doc]

**Table S2: Literature review for infectivity of fresh *Plasmodium knowlesi* sporozoites introduced by direct inoculation or by mosquito bite.**

| **Article** | **# of Bites or # of Sporozoites** | **# Infected** | **# Not infected** | **Prepatent period (days)** |
| --- | --- | --- | --- | --- |
| Cochrane et al. [37] | 2x10 ^3^ SPZ  (fresh SPZs) | 3/3 | 0 | 7 days |
| Butcher GA, Cohen S. Antigenic variation and protective immunity in *Plasmodium knowlesi* malaria. Immunology. 1972;23:503-21 | 1x10^2^-10^4^ SPZ | 4/4 | 0 | 3-4 days |
| Sullivan et al. [38] | 8 bites | 1/1 | 0 | 7 days |
|  | 1.1x10^6^ SPZ | 1/1 | 0 | 6 days |
| Collins et al. [39]  ***+ = Fresh SPZs***  ****= Frozen SPZs 7 Days – 11 Years***  ***(M. mulatta)***  *****= Frozen SPZs 11 Years***  ***(S. boliviensis)*** | 50 SPZ**^+^** | 6/6 | 0 | 13 days  (11-16) |
|  | 5.2x10^4^ SPZ* | 1/1 | 0 | 7 days |
|  | 5.4x10^4^ SPZ* | 1/1 | 0 | 6 days |
|  | 1.25x10^5^ SPZ* | 1/1 | 0 | 8 days |
|  | 1.25x10^5^ SPZ* | 1/1 | 0 | 10 days |
|  | 4.5x10^4^ SPZ* | 1/1 | 0 | 7 days |
|  | 45 SPZ ** | 0/1 | 1/1 | N/A |
|  | 450 SPZ** | 1/1 | 0 | 13 days |
|  | 4.5x10^3^ SPZ** | 1/1 | 0 | 10 days |
|  | 4.5x10^4^ SPZ** | 1/1 | 0 | 12 days |
